# In-Vitro and In-Silico Investigation for the Spent-Coffee Bioactive Phenolics as a Promising Aflatoxins Production Inhibitor

**DOI:** 10.3390/toxins15030225

**Published:** 2023-03-16

**Authors:** Amr Farouk, Tawfiq Alsulami, Hatem S. Ali, Ahmed Noah Badr

**Affiliations:** 1 Flavor and Aroma Chemistry Department, National Research Centre, Dokki, Cairo 12622, Egypt; 2 Food Science & Nutrition Department, College of Food and Agricultural Sciences, King Saud University, Riyadh 11451, Saudi Arabia; 3 Food Technology Department, National Research Centre, Dokki, Cairo 12622, Egypt; 4 Food Toxicology and Contaminants Department, National Research Centre, Dokki, Cairo 12622, Egypt

**Keywords:** aflatoxin reduction, antifungal, enzyme-docking, molecular dynamic, oxidative reactions, phenolic compounds, spent-coffee grounds

## Abstract

Aflatoxin, is a naturally occurring polyketide generated by *Aspergillus flavus* via biosynthetic pathways, including polyketide synthase (PKS) and non-ribosomal enzymes. The in vitro analysis supported by molecular dynamics (MD) techniques was used to examine the antifungal and anti-aflatoxigenic activity of spent coffee grounds (SCGs) methanol extract. The High-Performance Liquid Chromatography results revealed the presence of 15 phenolic acids and five flavonoids. (R)-(+)-Rosmarinic acid (176.43 ± 2.41 µg/g) was the predominant of the detected acids, followed by gallic acid (34.83 ± 1.05 µg/g). At the same time, apigenin-7-glucoside is the dominant flavonoid in the SCGs extract by 1717.05 ± 5.76 µg/g, and naringin (97.27 ± 1.97 µg/g) comes next. The antifungal and anti-aflatoxigenic activity of the SCGs extracts was 380 µL/mL and 460 µL/mL, respectively. The SGGs’ effect of inhibiting five *Aspergillus* strains’ growth on the agar media ranged between 12.81 ± 1.71 to 15.64 ± 1.08 mm by two diffusion assays. Molecular docking results confirmed the inhibitory action of different phenolics and flavonoids on the PKS and NPS key enzymes of the aflatoxin biosynthetic mechanism. The SCGs extract components with the highest free binding energy, naringin (−9.1 kcal/mL) and apigenin 7-glucoside (−9.1 kcal/mol), were subjected to an MD simulation study. The computational results infer the stabilizing effects on the enzymes upon ligand binding led to the impairment in its functionality. The current study represents a novel attempt to assess the anti aflatoxins mechanism of phenolics and flavonoids targeting PKS and NPS via computational approaches compared to in-vitro assays.

## 1. Introduction

Mycotoxicosis can lead to mortality caused by the secondary metabolites that fungi produce, known as mycotoxins, contaminating food commodities and feedstuffs in humans and animals. The impact of mycotoxins on gut health and gut microbiota was reported as harmful in previous studies, where several types of gut flora with health-aiding properties are lost [1]. The incidence of mycotoxin contamination is further increased by continuous global warming. Climate change is connected with determining dominant fungi and the main mycotoxin identified in some areas [2].

The *Aspergillus* fungi produce a category of mycotoxins known as aflatoxins. Four types of aflatoxins are dominant; they are known as aflatoxin B_1_ (AFB_1_), aflatoxin B_2_ (AFB_2_), aflatoxin G_1_ (AFG_1_), and aflatoxin G_2_ (AFG_2_). AFB_1_ is the most potent hepatocarcinogen identified, and the International Agency for Research on Cancer classifies it as a Group I carcinogen [3]. On the other hand, acute aflatoxicosis may cause stomach discomfort, vomiting, edema, and death [4]. Additionally, several animal [1] and bird diseases [5] were reported to be linked with aflatoxin contamination. Therefore, the abovementioned hazards require urgent efforts to avoid health and food supply risks.

Various strategies were utilized to overcome the contamination by aflatoxins (AFs) in food commodities, classified into chemical or biological strategies. Chemical approaches included synthetic chemicals such as ammonia, hypochlorite, and clays [6]. These applications are considered more hazardous with a possibility of risk for chemical residues [7]. Moreover, not all cases can be safe and free of aflatoxin contamination. At the same time, biological strategies depend on probiotic or lactic acid bacteria to bind aflatoxins to the cell wall components and decrease their toxicity [8]. This technique is considered safer and could provide some benefits such as adding value to the final product. The novel source of bioactive components was agro-industrial by-products, which are rich in phenolic, flavonoids, antioxidants, sterols, and polysaccharides [9]. Several extracts obtained from agro-industrial by-products were evaluated to decrease the mycotoxin hazard. 

For example, the extract gained from grape by-products was utilized to inhibit ochratoxin in simulated media. Otherwise, the oil extracted from pomegranate seeds [10] or mandarin was reported to be rich in bioactive components with an ability to reduce mycotoxin production in liquid media. Other by-product extracts, including Moringa, bottle gourd, and prickly pear [11,12], effectively reduce aflatoxins.

Spent coffee grounds (SCGs) are an agro-industrial by-product rich in phenolic compounds. Six million tons of SCGs are produced annually as a by-product of brewing [13]. Both phenolic and non-phenolic acids are predominant in the SCGs composition [14]. Phenolic compounds are beneficial and known to be antibacterial, antioxidant, anti-mutagenic, anti-inflammatory, and anti-allergenic [15]. Regarding the study by Loi et al. [16], phenolic compounds can mainly affect mycotoxigenic fungi growth resulting in mycotoxin secretion reduction. Other recent investigations pointed out that phenolic compounds are anti-mycotoxigenic agents with efficacy in aflatoxin reduction produced by their related fungi.

Determining the cytotoxicity, antifungal, and anti-AFs effects of the SCGs methanolic extract and interpreting its mode of action were the goals of the current investigation. To our knowledge, few studies have examined how natural ingredients work to inhibit the release of AFs. The molecular dynamics (MD) and docking of the phenolic SCG compounds regarding the AFs-producing enzymes have not been discussed in any studies. The current study focused on polyketide synthase and non-ribosomal peptide synthase, two large multimodular vital enzymes involved in the biosynthesis of polyketide and peptide products as secondary metabolites in AFs biosynthesis, to elucidate the anti-AFs action of SCGs phenolics at a molecular level [17]. The MD simulations also looked into how these supramolecular complexes affected the dynamics of the targeted proteins, which may have helped or hindered their ability to perform biologically.

## 2. Results

### 2.1. Characterization of SCGs Phenolic and Flavonoids

The results of the SCGs analysis for the presence of phenolic compounds reflected a wealth of content of phenolic acids, alkaloids, and flavonoid compounds (Table 1). Regarding phenolic acids, 15 compounds exist in the extract, where (R)-(+)-rosmarinic acid (176.43 ± 2.41 µg/g) was shown by the majority content of the detected acids, followed by gallic acid (34.83 ± 1.05 µg/g) and sinapic acid (11.9 ± 0.94 µg/g). Additionally, chlorogenic and caffeic acids existed significantly; 9.31 ± 0.94 and 8.58 ± 0.94 µg/g, respectively. Five compounds related to the flavonoid content of the SCGs are recorded to be present in the extract; apigenin-7-glucoside is the dominant flavonoid in the SCGs extract by 1717.05 ± 5.76 µg/g, while naringin (97.27 ± 1.97 µg/g) comes next. Three flavonoids exist, including catechin, epicatechin, and chrysin compounds. Caffeine is recorded as considerably high for the alkaloid content of the SCGs extract obtained by aqueous methanol (1592.82 ± 3.77 µg/g).

However, the yields and kinds of bioactive chemicals extracted from SCGs rely on several variables, including the species of coffee, the efficiency, and method of producing instant coffee, the storage conditions, and the extraction technique based on polar solvents. For example, the 60% methanol extract of roasted Arabica and blended coffee and SCGs showed the predominance of caffeine followed by chlorogenic acid [18]. In comparison, extraction with 100% MeOH by Ho et al. [19] revealed the presence of caffeine, 5- and 3-caffeoylquinic acid, quinic acid, vanillic acid, caffeic acid, epicatechin, catechin, ferulic acid, p-coumaric acid, p-hydroxybenzoic acid, and gallic acid as the major components of the phenolic extract of Ethiopian Yirgacheffe coffee. According to Zengin et al. [20], the total amount of phenolics in SCGs based on the solvent type was arranged in the following order; EtOH:H_2_O > MeOH:H_2_O > MeOH > H_2_O, with caffeine, chlorogenic, gallic, vanillic, and caffeic acids as the predominant. Therefore, except for caffeine, quantitative differences could be observed in the current study compared to the literature and among the published studies. However, the identified predominants have a typical qualitative pattern.

### 2.2. Cytotoxic Impact of SCGs Methanol Extract

Table 2 shows how the methanol extract affected the viability of human healthy oral epithelial (OEC), human colon carcinoma (HCT-116), and liver cancer (HepG2) cell lines. The HepG2 and HCT-116, cell viability percentages, were lower after treatment with the SCG extract than with the untreated OEC cell line, indicating the selectivity of the examined extract. For the MTT 3-(4,5-Dimethylthiazol-2-yl)-2,5 Diphenyltetrazolium Bromide (MTT assay) and the Sulforhodamine B colorimetric assay (SRB), the selectivity index (SI) was 2.30 and 3.12, respectively (Table 2). In general, the extract of SCGs demonstrated moderate cytotoxicity against the studied cells. The results from the MTT and SRB assays were similar in terms of their values. An SI value of (<1) indicates that the sample may be hazardous and cannot be utilized to make natural medicines, which is not the case in the current study. Compared to the positive control (Cisplatin), the cytotoxicity of SCGs methanol extract was lower.

The present findings revealed better activity for the SCGs methanol extract than that found by ethanolic extract in the previous study [21], where the treatment of HaCaT cells with extract at concentrations lower than 3 mg/mL did not affect cell viability. Meanwhile, the results of the current study agreed with the findings of Angeloni et al. [22], who extracted SCGs with H_2_O, MeOH, MeOH:H_2_O (50:50), and EtOH: H_2_O (30:70) and found no cytotoxicity up to 100 μg/mL, and some activity at 200 μg/mL on BV-2 microglial cells. In contrast, the isopropanol extract of SCGs showed higher cytotoxic activity than the present study’s findings [23].

### 2.3. Antifungal of the SCG-Extract using Diffusion Assays

The high content of the SCG extract of phenolic compounds led the authors to investigate its application as anti-aflatoxigenic material. Therefore, they evaluated five identified strains of *Aspergillus* fungi (Table 3) for the impact of phenolic extract. The data reflected a considerable inhibition effect of the SCG extract using two assays of evaluation (disk and well-diffusion assays). The result demonstrates more sensitivity of the strain *Aspergillus* fumigatus, which is recorded as more inhibition. The inhibition effect of the SCGs is apparent for the values recorded using the disk diffusion assay; these results are emphasized by the data recorded in the well-diffusion assay. Moreover, the minimal fungicidal concentration of the SCG extract against the applied strains of *Aspergillus* fungi ranges between 380 and 460 µg/mL. These results clarify the expected potency of the SCG by its application in antifungal products and as fungistatic material against toxigenic fungi.

Plants synthesize secondary metabolites (phytochemicals) to protect themselves from hazardous conditions [24]. Phytochemicals possess antimicrobial activity and may protect humans and animals against illnesses and diseases caused by microbes and their related toxins [7,25]. Phenolic compounds are recorded to efficiently suppress the fungal infection during the post-harvest stage [26], which may affect the mycotoxins-producing process on the stored commodities [16]. Herbal plants with antioxidant properties have antifungal and anti-mycotoxigenic activities [27]. The antigenotoxicity of plant extracts was tested against the genotoxicity caused by the AFB_1_. The Vitotox and Ames tests showed that most applied plant extracts had a medium to strong capacity to prevent genetic changes.

### 2.4. Estimation of the Anti-Aflatoxigenic SCG-Extract Effect

**Figure 1:**
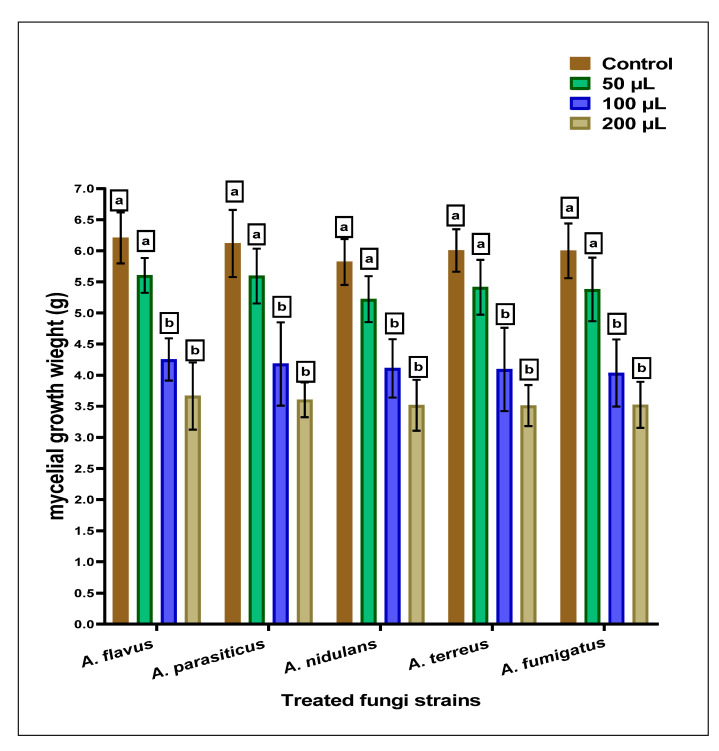
The anti-*Aspergilli* effect of methanolic SCGs extract represented mycelia growth inhibition using different concentrations than the growth of fungal strains (the control). For each concentration of treatment, the results with the same superscript letter (^a,b^) are non-significantly different.

The anti-*Aspergillus* and anti-aflatoxigenic properties of the SCG, which are determined using simulated growth media, are reflected by moderate efficiency against the applied strains of *Aspergillus* species. The result of inhibition against fungal strains is represented in Figure 1. The mycelial growth inhibition was raised by increasing the concentration in the SCG extract’s growth media. A little more sensitivity was recorded for applying SCGs extract against *Aspergillus fumigatus* fungi.

**Figure 2:**
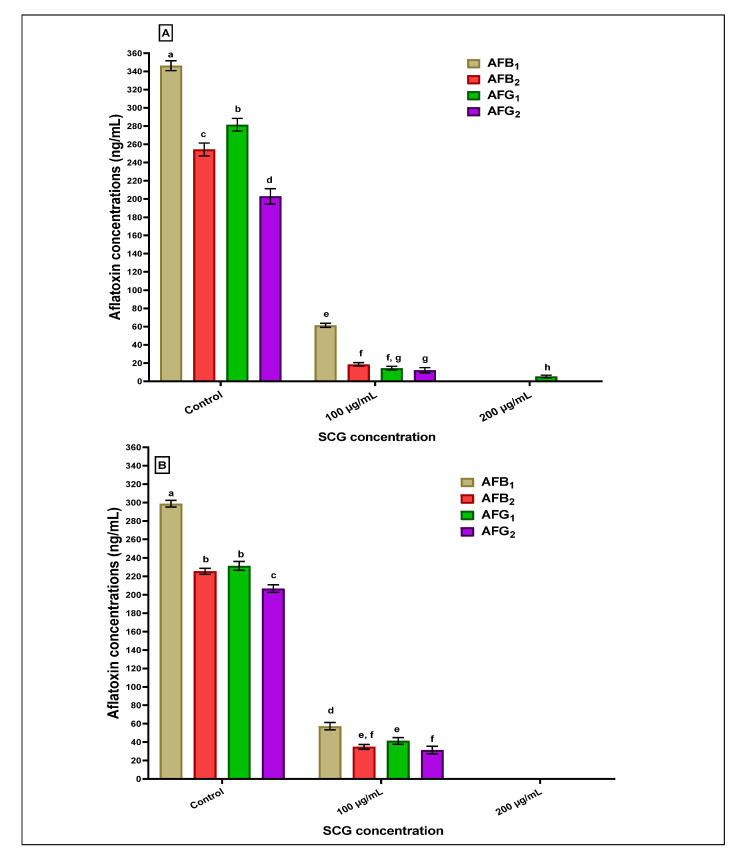
The anti-aflatoxigenic effect of methanolic SCGs extract was estimated as a reduction in aflatoxin production concentrations in liquid media. (**A**): aflatoxins reduction by the SCGs extract in media contained produced *A. flavus* strain. (**B**): aflatoxins reduction by the SCGs extract in media produced *A. parasiticus* strain; the results with different superscript (^a,b,c,d,e,f,g,h^) letters were recorded as significant differences for the data represented in each figure.

Figure 2 reflects the potency of the SCGs extract concerning the reduction estimated for the Afs production in liquid media by two producing strains of *Aspergillus* fungi (*A. flavus* and *A. parasiticus*). Inserting 100 µg SCG extracts/mL media into liquid media containing the A. flavus spores leads to a high reduction in aflatoxin concentrations of AFB_1_, AFB_2_, AFG_1_, and AFG_2_ produced in media (Figure 2A). This reduction entirely occurred for the AFB_1_, AFB_2_, and AFG_2_ by increasing the SCG applied concentration to 200 µg of SCGs extracts/mL media. In contrast, the AFG_1_ was found in traces. Concerning the exact estimations for Afs in liquid growth media of A. parasiticus, the result reflects the complete reduction at 200 µg of SCGs extracts/mL media. The decline in AFB1, _AFB2_, AFG_1_, and AFG_2_ using 100 µg of SCGs extracts/mL media into liquid growth media showed a lower reduction than that recorded for Afs at the same concentration on the *A. flavus* strain (Figure 2B).

Phenolic acids are found naturally in plant extracts and have been shown to limit fungal growth and mycotoxin formation in *Aspergillus* species [28]. However, considerable variability in the reaction of dangerous fungi to phenolic compounds is reported depending on the phenolic compound, the mycotoxin investigated, and the generating fungal strain [29]. Furthermore, it has been proposed that they might be due to experimental variations, with water stress being particularly crucial [30]. Previous research investigated how phenolic acids effectively inhibited *Fusarium* fungal growth and mycotoxin synthesis. Examining phenolic compounds’ effects on mycelial development and T-2 and HT-2 synthesis. Although most phenolic acids studied enhanced fungal biomass, the effects of phenolic acids on T-2 and HT-2 synthesis varied by phenolic acid type and strain [31]. Numerous methods fail to detoxify mycotoxins entirely and leave behind harmful by-products.

It has been observed that plant extracts rich in phenolic compounds can be used as promising bioagents to suppress fungal growth or reduce mycotoxin production [32]. The type of phenolic acid and investigated strain had different impacts on T-2 and HT-2 synthesis. Ferulic acid inhibited T-2 production in both fungal species, but p-coumaric acid affected T-2 and HT-2 synthesis. In the kinetic investigation, a greater dosage of ferulic acid (1 mM) considerably inhibited *Fusarium* fungal growth. The impact of phenolic compounds on trichothecene genes and transcription was recorded, and the proposed mechanism for toxin production inhibition was previously discussed, indicating the need for more investigation [31].

At low concentrations, chlorogenic acid and its hydrolyzed derivative, caffeic acid, have demonstrated a significant anti-mycotoxin effect against different mycotoxins, including the AFB_1_ [33]. According to a transcriptomic investigation, caffeic acid inhibited the expression of crucial genes needed for *A. flavus* to produce aflatoxin. The *A. flavus* toxigenic growth may also be inhibited by ferulic acid, a methylated form of caffeic acid [34]. The mechanisms behind the antifungal and anti-mycotoxin activities of phenolic compounds have been studied by Ahmed et al. [32]. They include modifications to the fungal membrane that affect permeability and function, the decrease in oxidative stress and inhibition of oxidases, and the downregulation of the expression of crucial genes involved in mycotoxin production.

Pizzolitto et al. [35] referred to the functionality of phenol compounds as possessing a negative impact on the growth rate of *Aspergillus fungi*, particularly in the lag phase. They pointed out some properties of phenolic compounds, including hydrophobicity [36], to be linked with phenolic activity in microbial inhibition. They also refer to the relation between the compound concentration and the degree of fungal inhibition that occurred. The inhibitory effects of phenolic compounds on microbial growth may be joined to the phenolic structure that contained a hydroxyl group [37]. This chemical structure and distribution may affect cell growth due to its effect on cell membrane destabilization.

### 2.5. Molecular Docking Analysis

**Figure 3:**
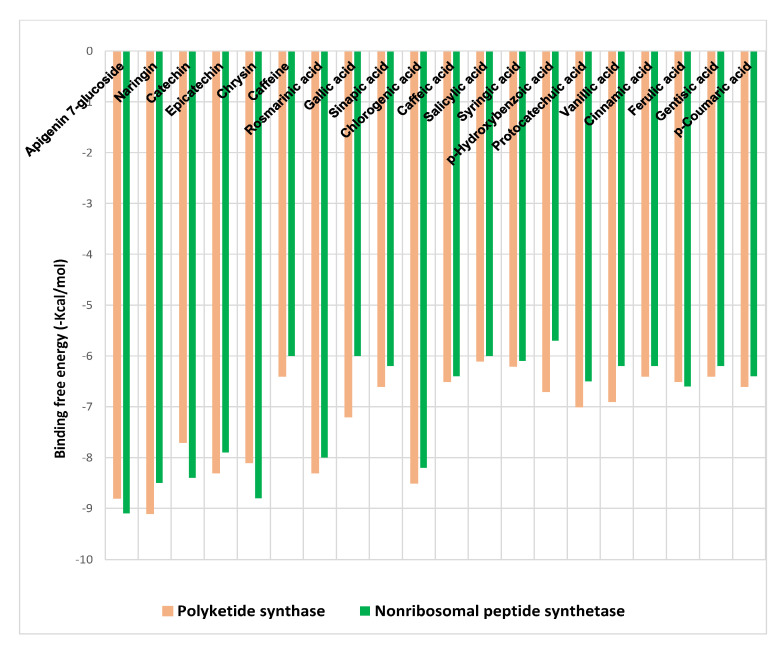
Binding free energy values were calculated through the molecular docking of the SCGs methanolic extract constituents and the receptors.

The binding free energies (∆G) for the phenolic acids and flavonoids of SCGs methanol extract ligands docked at polyketide synthase (PKS), and non-ribosomal peptide synthetase (NPS) receptors are shown in Figure 3, revealing the best poses obtained in the molecular docking analyses. The significance of the interaction between the receptor and the ligand with potential activity increases with decreasing ΔG.

Generally, flavonoids displayed higher binding affinities with high docking scores, especially for naringin (−9.1 kcal/mol) and apigenin 7-glucoside (−8.8 kcal/mol) at PKS and for apigenin 7-glucoside (−9.1 kcal/mol) and chrysin (−8.8 kcal/mol) at NPS. On the other hand, chlorogenic acid showed the highest affinities among phenolic acids toward both receptors (−8.5 and −8.2 kcal/mol), followed by rosmarinic acid (−8.3 and −8 kcal/mol) for PKS and NPS, respectively (Figure 3).

**Figure 4:**
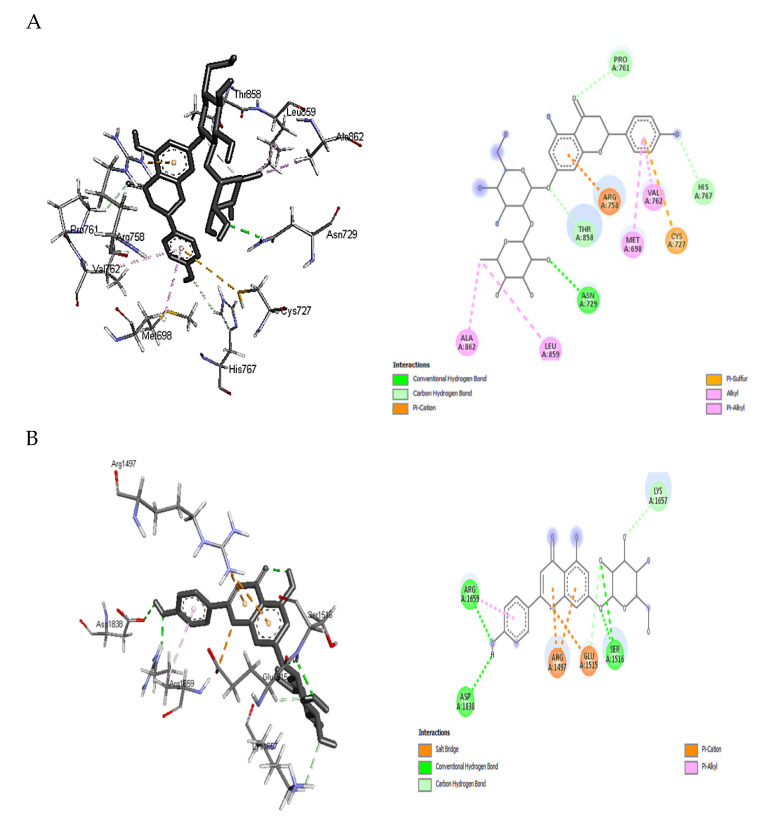
Insilico figures illustrate the interactions between the phenolic compounds and synthatase enzymes of aflatoxins. (**A**) the interaction happened between naringin with polyketide synthase. (**B**) the interaction happened between Apigenin 7-glucoside with non-ribosomal peptide synthetase.

Based on the binding free energy values discussed above, docking details of naringin and apigenin 7-glucoside toward PKS and NPS as the highest receptor-ligand binding positions scores were illustrated in Figure 4A,B. The higher binding affinity of naringin with PKS (−9.1 kcal/mol) is attributed to the conventional hydrogen bonding and carbon-hydrogen interaction formed with ASN A:729, PRO A:761, HIS A:767, and THR A:858. In addition, π -sulfur, alkyl, and π -alkyl hydrophobic interactions were also observed with CYS A:727, ALA A:862, LEU A:859, VAL A:762, and MET A:698. Hydrophobic contacts dominate interactions in protein-ligand complexes. The interactions between an aromatic or aliphatic carbon from the ligand and a chlorine or fluorine atom from the protein were the second most frequent hydrophobic connections, followed by interactions between a sulfur atom from the amino acid moiety and an aromatic carbon from the ligand [38].

An aliphatic carbon In the receptor and an aromatic carbon In the ligand produce the majority of Interactions In this category, indicating that aromatic rings are frequently found in small molecule inhibitors. The benzene ring system is the most prevalent aromatic ring, and 76% of commercially available medications contain one or more aromatic rings. In protein structures, π-cation interactions have been thoroughly investigated. Gallivan and Dougherty [39] discovered that arginine side chains are more likely to make these connections than lysine and that π-cation interactions are rarely buried. Several mutation experiments have determined the interaction strength between a buried TRP and LYS, ARG, or HIS side chain from −0.8 to −0.5 kcal/mol. Finally, since an electron-deficient alkyl substituent frequently establishes direct contact instead of the cationic center, cation interaction with ARG A:758 may be considered a hydrogen-bonded system (Figure 4A).

Similar interactions could be observed between apigenin 7-glucoside and NPS residues than naringin, which explains the exact value of binding affinity. For example, conventional hydrogen bonds and carbon-hydrogen interaction were noticed with the moieties: ASP A:1838, ARG A:1659, SER A:1516, LYS A:1657, and GLU A:1515 (Figure 4B). The contact between a positively charged nitrogen and a negatively charged oxygen (i.e., salt bridge) was determined with GLU A:1515. Positive nitrogen from the protein and negative oxygen from the ligand resulted in twice as many salt bridge interactions as the opposite. The environment has a significant impact on how robust salt bridge interactions are. In particular, buried salt bridges can contribute to ligand binding [38]. Again, other hydrophobic interactions such as π -alkyl with ARG A:1659 and π -cation where the nitrogen came from the receptor (ARG A: 1497 of NPS) and the aromatic ring from the ligand (apigenin 7-glucoside) were observed in Figure 4B. Other interaction cites were provided as Supplementary Materials Figures S1–S5, which was less effective that these presented above.

To our knowledge, nothing in the literature concerning the in silico studies involved inhibiting the key enzymes responsible for aflatoxin biosynthesis using flavonoids or phenolic acids. However, a few studies have been published recently revealing the use of gingerol derivatives against the PT-domain of PKS-A [40], isoflavones against PKS [41], eugenol in nanoemulsion and verbenol-chemotype Zingiber officinale essential oil against *Aspergillus flavus* and aflatoxin B_1_ [17,42], mono- and sesquiterpenes of ginger oil in nanoemulsion form [43], and in vitro degradation of aflatoxins by a recombinant laccase from *Saccharomyces cerevisiae* [44]. The present study opens perspectives toward good predictions toward using different botanical and natural extracts which contain common phenolics and flavonoids investigated and examined during the in silico analysis. 

### 2.6. Validating Analysis of Receptor-Ligand Complexes Using MD Simulation

#### 2.6.1. PKS-Naringin

All-atom molecular dynamics simulations of receptor-ligand complexes of target proteins were run for 100 ns concerning the modeled structures of the target proteins to examine the impact of naringin binding on the targeted PKS. To investigate the inhibitory potential of naringin against PKS, different computations (structural, dynamical, and thermodynamic) were made on the observed trajectory. The backbone RMSD, RMSF, Rg, SASA, H-bond, and MM/PBSA analyses were used to evaluate the structural behavior of the target proteins in both their ligand-bound and unbound states.

**Figure 5:**
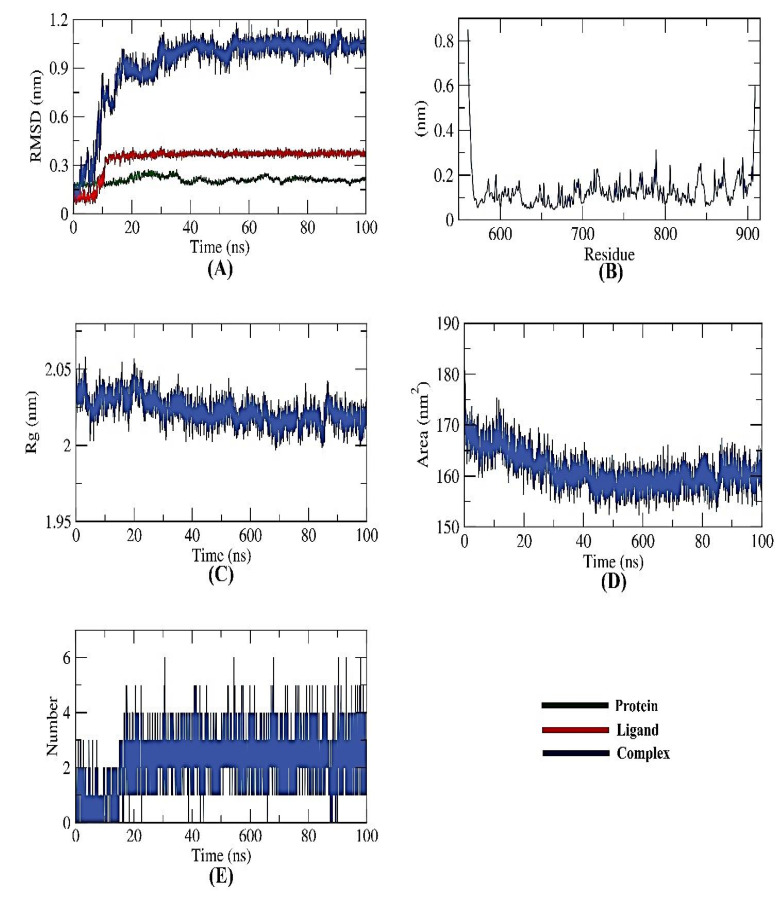
MD simulations of PKS-naringin complex: (**A**) RMSD, (**B**) RMSF, (**C**) Rg, (**D**) SASA, and (**E**) H-bond analysis.

To determine if the protein-ligand complex is stable in the presence of the receptor and ligand-bound state, the dynamic movements of atoms and conformational changes of backbone atoms were calculated using the RMSD (root-mean-square deviation) formula. The PKS, naringin, and complex have very low RMSD and no significant variations, indicating higher stability. Before displaying stability, the complex was unstable for ~17 ns (Figure 5A). The more stable protein structures are those with lower RMSD values, and vice versa [18]. Likewise, the residues’ RMSF (root mean square fluctuation) was observed to find fluctuation from their time-averaged position during the simulation. The number of residues defines how flexible the proteins are.

As presented in Figure 5B, it could be seen that the fluctuation seems to be neutral, emphasizing no effects of the ligand binding on the residue positioning. Similar to RMSD, the radius of gyration (Rg) measures protein compactness that scales inversely. The protein’s compactness varies depending on whether it is coupled to the ligand. Lower fluctuation across the simulation time indicates a system’s better compactness and consequent stability. It was discovered that the Rg of the PKS-naringin complex was somewhat lower than in the initial period (Figure 5C). The interaction of the PKS-naringin complex with the surrounding solvents was also examined using a solvent-accessible surface area (SASA) over 100 ns. SASA values can precisely predict the conformational changes after binding any complex components.

Interestingly, the PKS decreased its surface area over 100 ns of simulation, as depicted in Figure 5D, and provided a reasonably constant SASA value. A protein-ligand combination must have hydrogen bonds for the structure to be stable. Five hydrogen bonds between the protein’s largest number of conformations and the ligand were shown to form (Figure 5E).

**Figure 6:**
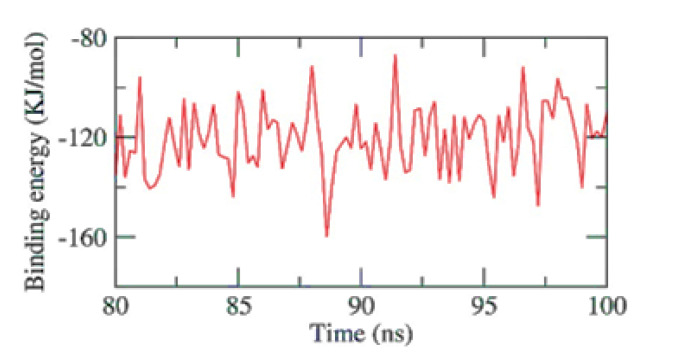
MM-PBSA study of the PKS-Naringin complex.

The stability of an inhibitor’s binding to an enzyme is highly connected with the inhibitor’s capacity to inhibit the enzyme in enzyme-inhibitor systems. Therefore, when assessing the MD simulation studies of the inhibitors, it is essential to consider the binding of free energy to the active sites of the target proteins. The current study predicted the binding free energies for the receptor-ligand complex systems, i.e., PKS-naringin, using MD simulations in conjunction with the MM/PBSA technique. Naringin’s binding affinity profiles against PKS were employed to determine the optimal inhibitory action’s selectivity (Figure 6). The MM/PBSA method was used to determine the binding free energy of the final 20 ns of the MD production run with an interval of 100 ps using MD trajectories. The output files from g mmpbsa were also used to compute the average free binding energy and its standard deviation/error using the MmPbStat.py script. The PKS protein displayed a binding free energy of −120 KJ/mol with the naringin.

#### 2.6.2. NPS-Apigenin 7-Glucoside

**Figure 7:**
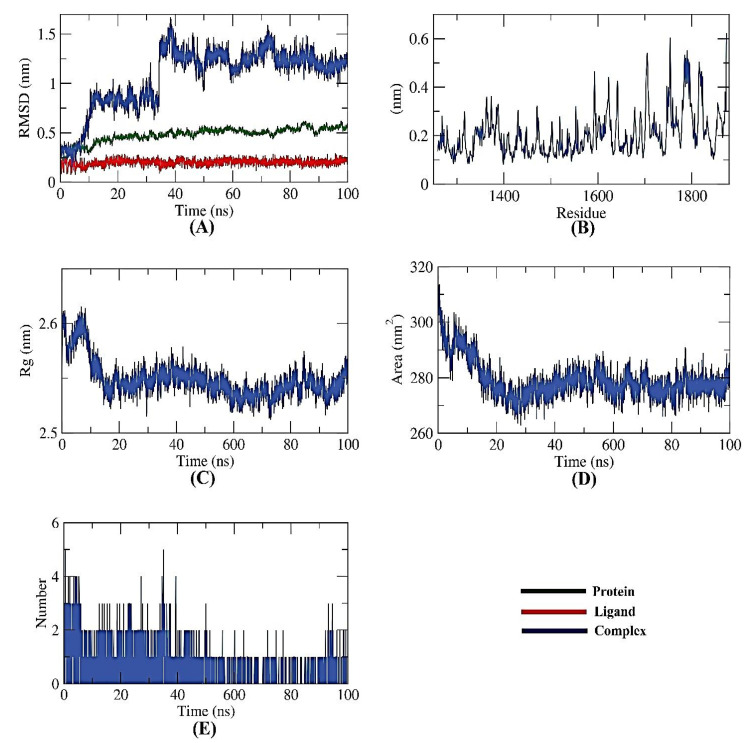
MD simulations of NPS-Apigenin 7-glucoside complex: (**A**) RMSD, (**B**) RMSF, (**C**) Rg, (**D**) SASA, and (**E**) H-bond analysis.

Several MD simulation studies looked into the dynamic conformational changes of the apigenin 7-glucoside-NPS complex. Initially, it was thought that RMSD would reveal the stability of the complex for a time period of 100 ns in both the initial and bonding phases. Interestingly, the apigenin 7-glucoside-NPS complex was unstable for the first ~10 ns before becoming stable for the next ~40 ns with minimal variation (Figure 7A). Second, using the RMSF, the complex’s flexibility was investigated at the atomic level. The regions in the target (NPS) that fluctuated after binding were made visible by the RMSF computation. The protein becomes slightly more flexible in the 1700–1800 residue range upon ligand interaction (Figure 7B). The Rg also served as a representation of the apigenin 7-glucoside-NPS compactness. As shown in Figure 7C, the complex’s Rg was lower at the end of the 100 ns than at the beginning, demonstrating the system’s stability and compactness.

Additionally, SASA was used to examine the interactions over 100 ns between the apigenin 7-glucoside-NPS complex and the surrounding solvents. At the end of the simulation period, the NPS showed a decline in its surface area, indicating a SASA value that was relatively stable (Figure 7D). A protein-ligand combination must have hydrogen bonds for the structure to be stable. It was observed that the highest number of conformations of the protein formed up to three hydrogen bonds with the ligand (Figure 7E).

**Figure 8:**
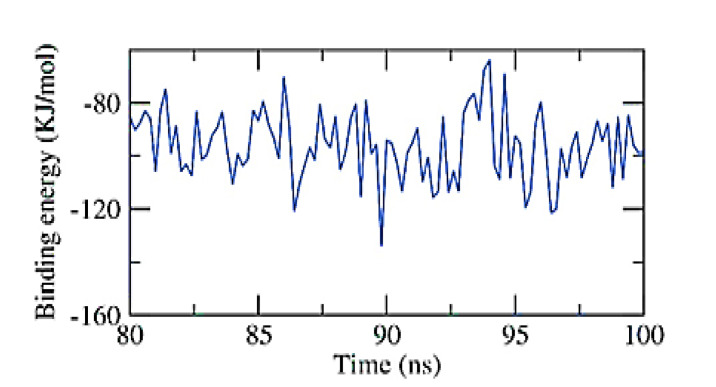
MM-PBSA study of the NPS-Apigenin 7-glucoside complex.

According to the MM/PBSA approach, the exact binding free energy of the NPS-Apigenin 7-glucoside complex was investigated during the final 20 ns of the MD simulation experiment, using a 100 ps interval from the MD trajectories. Additionally, the MmPbSaStat.py script was used to calculate the output files’ standard deviation and/or standard error from the g mmpbsa and the average binding free energy. Figure 8 shows that the binding free energy of apigenin 7-glucoside to the NPS was −96 KJ/mol. The gathered information demonstrated that the NPS-Apigenin 7-glucoside complex binds with the appropriate kinetics, energy, and structural modifications.

## 3. Conclusions

Although several previous works of literature have attempted to provide speculations about the role of phenolic compounds as a factor participating in inhibiting fungal growth, they have not provided concrete evidence, such as bioinformatic studies. The current study was interested in explaining the behavior of fungi that grew in media rich in phenolic extracts compared to fungi in the control one. Preliminary results indicated that fungal growth was significantly affected by coffee-spent ground extract in the growth media as a source of polyphenols. The laboratory study’s in-vitro results suggested that the fungus’s development was affected by the appearance of inhibition zones in the diffused agar environment or a decrease in mycelial growth in the liquid growth environments. The results were supported by evaluating phenolic compounds’ binding impact by molecular docking or molecular dynamic. The current study represents a novel attempt to assess the anti-aflatoxin mechanism of phenolics and flavonoids from SCGs methanol extract targeting PKS and NPS through in-silico techniques. Flavonoids, especially apigenin 7-glucoside, and naringin, showed higher activity among all identified extract constituents. The antifungal, anti-aflatoxigenic activity and inhibition of five Aspergillus strains’ growth by SCGs methanol extracts were confirmed by molecular docking results. According to the computational results of the MD simulations, the stabilizing effects on enzymes caused by ligand binding reduce their functioning.

## 4. Materials and Methods

### 4.1. Materials, Chemicals, and Microorganisms

Spent coffee was presented to the research team as an endowment from Misr-Cafe Company on the 10th of Ramadan Industrial City, Cairo, Egypt. The powder was milled to close micronized granules (40 mesh) for the extraction and application steps. The powder was dried immediately (40 ± 1 °C) using a Hot-air oven (Model ED 56, Binder GmbH, 78532 Tuttlingen, Germany) until completely dried. Five strains of toxigenic fungi identified as *A. flavus* ITEM 698, *A. parasiticus* ATCC 15517, *Aspergillus nidulans* ATCC 26209, *Aspergillus terreus* ATCC 1012, and *A. fumigatus* ATCC 1022 for the antifungal susceptibility investigations.

### 4.2. Preparation of Spent Coffee Extract

The extraction was performed per the optimized procedure described by Mussatto et al. [45]. In brief, the SCGs were extracted using aqueous methanol (60%) in a solvent/solid ratio of 40 mL/g of SCGs over 90 min at a temperature between 60 and 65 °C in a water bath with mechanical Agitation. At the time-ended, the solvent-slurry was centrifuged at 2500× *g* (20 min/4 °C), where the supernatant was filtered through a 0.22 µm filter. The calculations were performed by quantifying and using the recovered volume of extract. The collected extract was stored darkly (at −20 °C) until the following evaluations.

### 4.3. Determination of Phenolic Acids and Flavonoids

The examination was conducted with an Acquity H class UPLC system fitted with a Waters Acquity PDA detector (Waters, Milford, MD, USA). The conditions and the properties of the column were the same as those of Stuper-Szablewska et al. [46]. The acetic acid solution in water (2%; pH of 2) was used as the chromatographic separation eluent and was included by acetonitrile (gradient solution). The concentrations of phenolic acids were measured at =320 and 280 nm with the assistance of external standards, and the detection limit was set at one nanogram.

### 4.4. Determination of the Cytotoxicity of SCGs

#### 4.4.1. Determination of the Cytotoxic Effect Using the Tetrazolium-Based (MTT) Assay

Human oral epithelial (OEC), colon (HCT-116), and liver cancer (HepG2) cell lines were cultured at a density of 1 × 104 cells/well (100 µL) in Dulbecco’s Modified Eagle Medium (DMEM) with antibiotics (10,000 U of penicillin and 10 mg of streptomycin in 0.9% saline) and 10% phosphate buffer saline serum (PBS). They were then kept at 37 °C and 5% CO_2_ for the following 24 h. A serially diluted extract was used to treat the OEC and HepG2 cells after 24 h, with concentrations ranging from 1000 to 0.01 µg/mL for OEC and 200 to 0.01 µg/mL, respectively. A positive control (Cisplatin) was used to compare concentrations ranging from 400 to 0.01 µg/mL. Then, 10 µL of a 12-mM MTT stock solution (5 mg/mL MTT in sterile PBS) was applied to each well. The MTT solution was removed after 4 h of incubation at 37 °C, and the precipitated purple formazan crystal was dissolved in dimethyl sulfoxide (DMSO) for 20 min. A 100 µL of an uncultured medium was combined with 10 µL of the MTT stock solution as a negative control. With a BMG LABTECH^®^-FLUOstar Omega microplate reader (Ortenberg, Germany), the absorbance was determined at 540 nm. The proportion of surviving cells was calculated as follows:

[(OD _sample_ − OD _blank_)/(OD _control_ − OD _blank_) *×* 100%] (1)

where OD _sample_: the optical density of the sample, OD _blank_: the optical density of the blank (DMSO), OD _control_: the optical density of the control.

The curve was illustrated based on the variation of the proportions of surviving cells according to concentrations, and IC_50_ was calculated using a sigmoidal curve obtained [23].

#### 4.4.2. Determination of the Cytotoxic Effect Using Sulforhodamine B (SRB) Assay

The SRB assay was used to test the extract’s cytotoxic effects on the HepG2, HCT, and OEC cell lines. In 96-well plates, aliquots of a 100 µL cell suspension (5 × 10^3^ cells) were incubated before being cultured in a complete medium for 24 h. A second aliquot of 100 µL of medium containing the extract or the positive control (Cisplatin) was used to treat the cells at concentrations ranging from 0.01 to 1000 µg/mL. Cells were fixed by changing the medium with 150 µL of 10% TCA and incubating at 4 °C for 1 h after 72 h of drug exposure. After eliminating the trichloroacetic acid (TCA) solution, the cells underwent five rounds of distilled water washing. Aliquots of a 70 µL SRB solution (0.4% *w*/*v*) were added and then incubated for 10 min in a dark environment at room temperature. The plates were air-dried overnight after being cleaned with 1% acetic acid three times. The protein-bound SRB stain was then dissolved in 150 µL of 10 mM tris aminomethane (TRIS), and the absorbance was determined at 540 nm using a BMG LABTECH^®^-FLUOstar Omega microplate reader (Ortenberg, Germany) [23].

#### 4.4.3. Determination of Selectivity Index (SI)

The selectivity index (SI) compares a sample’s toxic and actual bioactive concentrations. When used during in vivo treatment, the larger ratio of SI denotes a more potent and secure medication [18]. The following equation evaluated the SI value:

SI = IC_50_^No cancer cell^/IC_50_^Cancer cell^ (2)

### 4.5. Determination of Antifungal Effects

#### 4.5.1. Determination of SCG Minimal Inhibition Concentration

The CLSI reference method for broth dilution antifungal susceptibility testing of molds M27-A3 [47] was utilized to ascertain the extract’s minimal inhibitory concentration (MIC) using Mueller–Hinton media (MHB). In brief, serial dilutions of the stock extract (ranging from 10 to 1000 µg/mL) were made with broth media where the test pathogens were applied at 0.4 × 10^3^ CFU/mL. Capped tubes were incubated (28 °C/72 h), and the evaluation performed included positive controls (media contained fungi only), negative controls (culture media free of fungi), and extract control (media contained solvent extract). The minimal inhibitory concentration (MIC) was determined to be the lowest concentration that effectively stopped the growth of fungi through visual inspection. 

To determine the in-vitro fungicidal activity of the methanolic SCGs extract, 10 µL of the wells that showed complete inhibition (100% inhibition; optically clear well), 10 µL of the final positive well (well with growth just before the clear well) and 10 µL of the controlled growth well were streaked onto Sabouraud dextrose agar plates. The clear plates with no growth for the fungi strain indicate the efficiency as the minimal fungicidal concentration.

#### 4.5.2. Determination of Disk and Well Diffusion Assays

Spore suspensions of tested fungal strains were prepared in tween-water (1%) according to the methodology described in Shehata et al. [48]. The concentration of the spores was adjusted, using a hemocytometer slide, to 10^3^ CFU/mL. The test was performed according to the CLSI M27-A3, using Alastruey-Izquierdo et al. [49] with slight modification. Mueller–Hinton agar media plates were streaked evenly with a swab dipped into the standardized spore suspension.

Disks containing the MSCG (0.4 mg/mL) were spread to the inoculated plate surface. Sterile disks were loaded with Nystatin (0.1 mg/mL) and applied as a standard reference for positive control plates, while negative ones contained disks loaded by the DMSO. Plates were incubated (28 °C/4 days) to permit fungal growth, where zones of inhibition diameters (ZID) were measured in millimeters. The same steps were repeated for the well-diffusion assay, where wells were filled using 400 μg/mL SCG-extract. In control positive plates, nystatin (100 μg/mL) was loaded into the wells, where the negative control well was loaded by the DMSO. The plates were incubated (28 °C/4 days) to give access to fungal growth.

### 4.6. The Antifungal SCG Influence Using Simulated Liquid Media

The inhibition effect of the SCG extract was determined at three concentration levels (50, 100, and 200 µg/mL media) using a simulated media. The inhibition effect was calculated as weight loss of the dried fungal mycelia of the treated strain compared to the controlled growth [11]. In conical flasks of 500 mL, a volume of Czapek–Dox broth media (CZB) equal to 150 mL was autoclaved to evaluate the SCG-extract impact on the fungal growth weight of mycelia. The strains were cultured individually (10^5^ CFU/mL) into flasks with or without the SCGs-extract in media. Each fungus’s inhibition in mycelia growth was calculated after liquid-media filtration on known-weight filter paper. The filter papers were dried in a hot air oven until the constant weight; the following equation was utilized to calculate the inhibition against the controlled growth of the same fungi.

% MI = ((Wc − Wt)/Wc) × 100(3)

where %MI: mycelial inhibition ratio, Wc: mycelia weight of the control flask, and Wt: mycelia weight of the treated flask

### 4.7. Estimation of the Changes in Aflatoxin Production

The strains of *A. flavus* and *A. parasiticus* were previously known as AFs producers. The toxin concentrations in filtrated media after incubation (28 °C/12 days) were evaluated in the extract collected from simulated media compared to the control [50]. Briefly, two groups of conical flasks of 0.5 L were sterilized (containing 200 mL CZB media). For each strain of fungi, a group consisting of SCGs-extract flasks (100 µL, 200 µL) and control flasks (without extract). The SCGs-extract was filtered using a micro-filter syringe (0.22 µm) before inoculating to the flasks, while the flasks were used as control growth, and normal AFs production was applied without SCGs-extract. The sterile media of all flasks (control and SCGs-extract) were inoculated with 10^5^ CFU/mL using fungal spore suspension of each group of fungi strains. The AFs were determined in an SCG-treated flask of *A. flavus* and *A. parasiticus* strains compared to the control of the same strains.

### 4.8. Mycotoxin Determination

Using the VICAM fluorometry method, the extractions of the media samples were subjected to aflatoxin quantification. In a nutshell, 25 mL of extracted media were combined with 5 g of NaCl, 50 mL of aqueous methanol (80%), homogenized in a lab for 1 min, and then filtered (Whatman paper no.1). A portion of the filtrate (5 mL) was refiltered after being diluted with 20 mL of deionized water. Aflatoxin-specific (AFB_1_) monoclonal antibodies were used to purify the 10 mL filtrate using VICAM immunoaffinity columns (VICAM Aflatest, MA, USA), after which it was washed with 10 mL of deionized water, and the aflatoxin was eluted with 1 mL of methanol. The eluted fraction was diluted twice with HPLC water, and the VICAM fluorometer was used to quantify it (VICAM Series 4EX Fluorometer). Everything was carried out following the manufacturer’s instructions. This methodology’s detection limit (0.1 ng/Kg) was previously validated [51].

### 4.9. Molecular Docking

Both polyketide synthase (A0A1R3RGK0) and non-ribosomal peptide synthase (A0A1R3RGK1) crystal structures were acquired from UniProt (accessed on 3 February 2022). Co-crystallized ligands and ions were removed and then protonated using the Pymol program (Ver. 2.5.1) to prepare it as a receptor. The MMFF94 force field was used by Avogadro Software (Version 1.2.0) to optimize the 3d structures of the ligands, which were downloaded from the PubChem database (http://pubchem.ncbi.nlm.nih.gov, accessed on 3–5 February 2022) [52]. A web-based software called CB-Dock, accessed from February 3 to 7 of 2022 (http://clab.labshare.cn/cb-dock/php/, accessed on 3–9 February 2022), was used to accomplish blind docking. Following submission, CB-Dock examined the input files and used MGLTools and OpenBabel to transform them into pdbqt-formatted files. CB-Dock then predicted the protein cavities, and their top N (*n* = 5 by default) centers and diameters were computed. The pdbqt files, each center, and size were sent to AutoDock Vina for docking. The final findings were shown after completing the N cycles of computation. The benchmarks performed by Liu et al. [53] showed success rates for top-ranking poses with RMSDs less than 2 Å from their location in the X-ray crystal structure. With Discovery Studio software Ver. 21.1.0.20298, CB-Dock surpassed other blind docking technologies in interface and visualization profiles for the best-docked complexes [53].

### 4.10. MD Simulation Methodology

The protein-ligand complexes were MD simulated using the Linux 5.4 package and GROMACS 2021.1. The PRODRG2 server created the ligand topologies, and the GROMOS96 54a7 forcefield was selected as the protein force field. All complexes were dissolved using water molecules with a simple point charge (SPC) in a rectangular container. Salt concentrations of 0.15 mol/L were set in all situations, and the appropriate Na+ and Cl- ions were introduced to the simulated system to make it electrically neutral. The steepest descent method performed 5000 iterations of energy minimization on all solvated systems. The MD simulation performed the production run, the NPT (constant number of particles, pressure, and temperature) series, and the NVT (constant number of particles, volume, and temperature) series. The simulation, performed on the NVT and NPT series at 300 K and 1 atm pressure for 300 ps, used the V-rescale thermostat and Parrinello–Rahman barostat. The simulation of molecular dynamics was completed after 100 ns at 300 K. The stability of the complexes under examination was then assessed by a comparative study that looked at the root mean square deviation (RMSD), root mean square fluctuation (RMSF), the radius of gyration (Rg), solvent accessible surface area (SASA), and hydrogen bonds. The Xmgrace program was used to plot the analyses [54].

### 4.11. Statistical Analysis

GraphPad Prism 7 was used for statistical data analyses (Graph Pad Software Inc., San Diego, CA, USA). The results were given as means with standard deviations (SD) based on at least three replicates. ANOVA was used to establish the significance of the difference between the mean values, and Duncan’s multiple range tests were computed (*p* = 0.05).

## Figures and Tables

**Table 1:** Contents of phenolic acids, flavonoids, and caffeine determined in SCGs methanol extract.

Phenolic Acid Contents of the SCG Extract			
Compound	Quantities (µg/g)	Compound	Quantities (µg/g)
Gallic acid	34.83 ± 1.05	Sinapic acid	11.9 ± 0.94
Protocatechuic acid	2.75 ± 0.41	(S)-(−)-Rosmarinic acid	0.7 ± 0.11
*p*-Hydroxybenzoic acid	5.42 ± 0.67	Ferulic acid	0.45 ± 0.08
Gentisic acid	0.38 ± 0.06	Salicylic acid	8.16 ± 0.73
Chlorogenic acid	9.31 ± 0.94	*p*-coumaric acid	0.22 ± 0.03
Caffeic acid	8.58 ± 0.56	Cinnamic acid	1.073 ± 0.55
Syringic acid	3.41 ± 0.41	(R)-(+)-Rosmarinic acid	176.43 ± 2.41
Vanillic acid	2.26 ± 0.04	-	-
Flavonoids contents of the SCG extract			
Compound	Quantities (µg/g)	Compound	Quantities (µg/g)
Catechin	18.78 ± 0.89		
Epicatechin	9.49 ± 1.02	Apigenin-7-glucoside	1717.05 ± 5.76
Naringin	97.27 ± 1.97	Chrysin	1.06 ± 0.03
Alkaloid contents of the SCG extract			
Compound		(µg/g)	
Caffeine		1592.82 ± 3.77	

The data were expressed as means ± SEM (where *n* = 3, LSD = 0.704, *p ≤* 0.05); SEM: standard error means; LSD: The least significant difference; SCGs: spent coffee grounds.

**Table 2:** Cytotoxic activity of SCGs extract against HepG2 and OEC cell lines using MTT and SRB assays.

Extract	Cell Lines	IC_50_ (μg/mL)	SI
Cisplatin	HepG2	66.69	1.31
	HCT-116	58.85	1.49
	OEC	87.67	-
SCG extract (MTT)	HepG2	341.3	2.30
	HCT-116	250.4	3.12
	OEC	784.3	-
SCG extract (SRB)	HepG2	347.1	1.94
	HCT-116	327.2	2.05
	OEC	672.3	-

For the MTT test: the value of the LSD was (4.542); R2 = 0.9912. For the SRB test: the value of the LSD was (3.139); R2 = 0.995; IC50: the half-maximal inhibitory concentration; SI: selectivity index.

**Table 3:** The antifungal activity of the SCGs extract using diffusion assays.

Fungi Strains	Disk Diffusion (ZID; mm)	Well Diffusion (ZID; mm)	MFC (µg/mL)
*Aspergillus flavus* ITEM 698	12.81 ± 1.71 ^c^	13.63 ± 2.05 ^b^	460
*Aspergillus parasiticus* ATCC 15517	13.57 ± 1.54 ^b,c^	14.21 ± 1.46 ^a,b^	420
*Aspergillus nidulans* ATCC 26209	14.05 ± 1.12 ^a,b^	14.34 ± 1.27 ^a,b^	380
*Aspergillus terreus* ATCC 1012	13.88 ± 1.41 ^b,c^	14.23 ± 1.02 ^a,b^	390
*Aspergillus fumigatus* ATCC 1022	15.64 ± 1.08 ^a^	15.02 ± 1.14 ^a^	380

The data were expressed as means ± SEM (where *n* = 3, SEM: standard error means). The results with the same superscript letter (^a,b,c^) of each column are non-significantly different.

## Data Availability

The data used to support the findings of this study are included in the article.

## Supplementary Materials

The following supporting information can be downloaded at: https://www.mdpi.com/article/10.3390/toxins15030225/s1, Figure S1: Interaction between Rosemarinic acid and Polyketide enzyme; Figure S2: Interaction between epicatechin and Polyketide enzyme; Figure S3: Interaction between Chlorogenic acid and Polyketide enzyme; Figure S4: Interaction between Chrysin phenolic compound and non-ribosomal enzyme. Figure S5: Interaction between catechin phenolic compound and non-ribosomal enzyme.
