# Supplementary material for: In-Vitro and In-Silico Investigation for the Spent-Coffee Bioactive Phenolics as a Promising Aflatoxins Production Inhibitor
Source: Toxins (Basel). 2023 Mar 16;15(3):225. doi: 10.3390/toxins15030225 (PMC10051990; doi:10.3390/toxins15030225)
Supplement: Supplementary file 1 [file toxins-15-00225-s001.zip › toxins-2269380-supplementary.pdf]

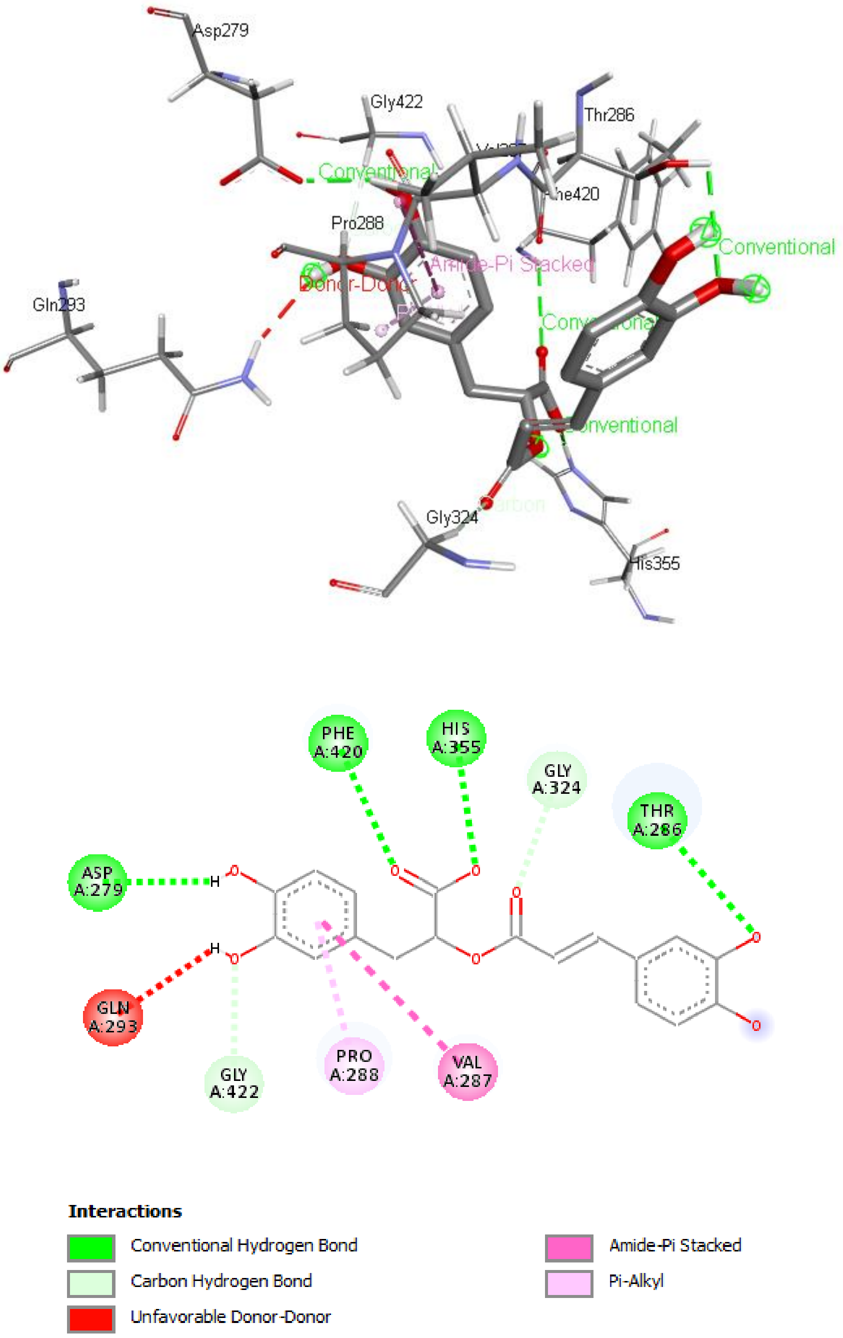

Figure S1. Interaction between Rosemarinic acid and Polyketide enzyme.

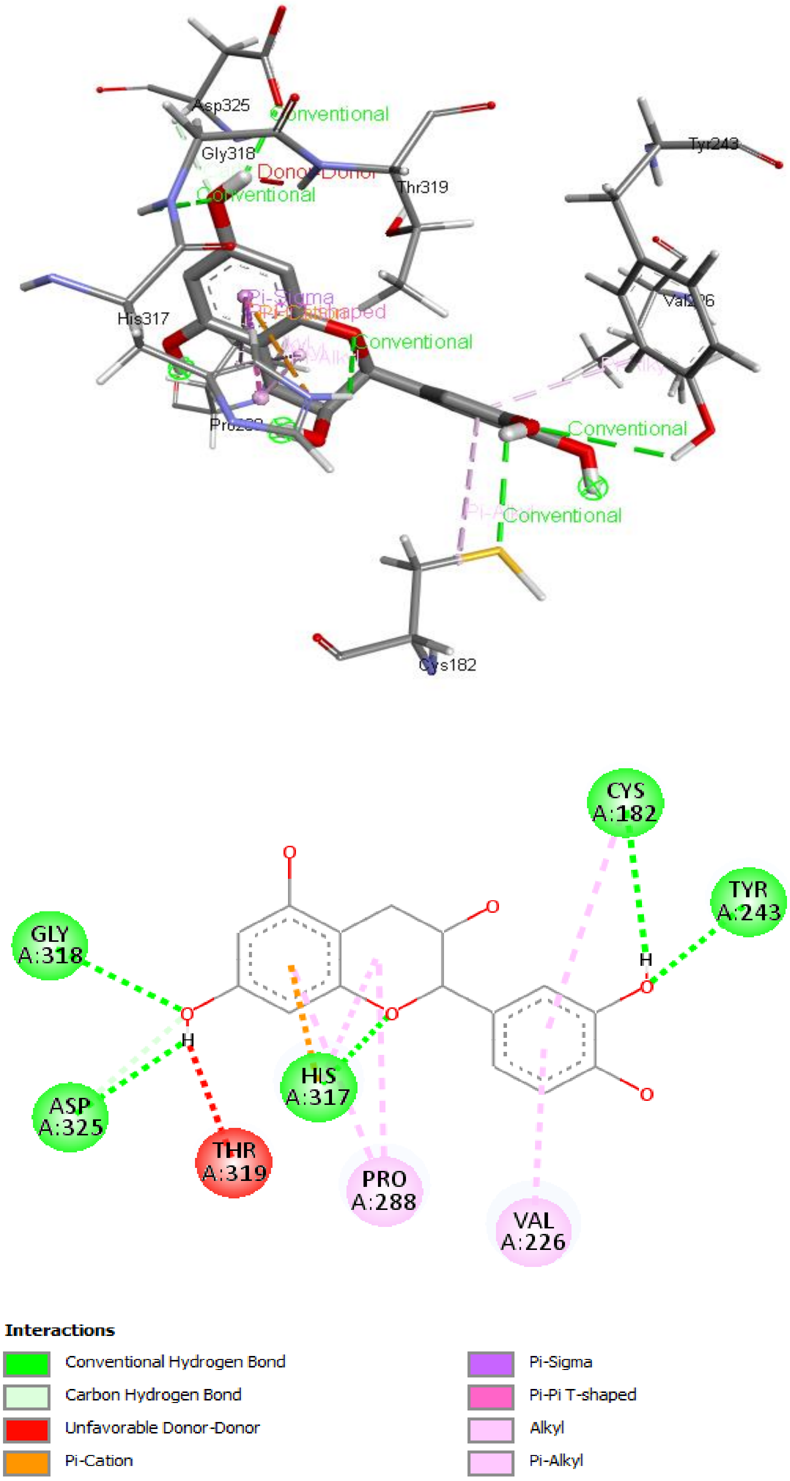

Figure S2. Interaction between epicatechin and Polyketide enzyme.

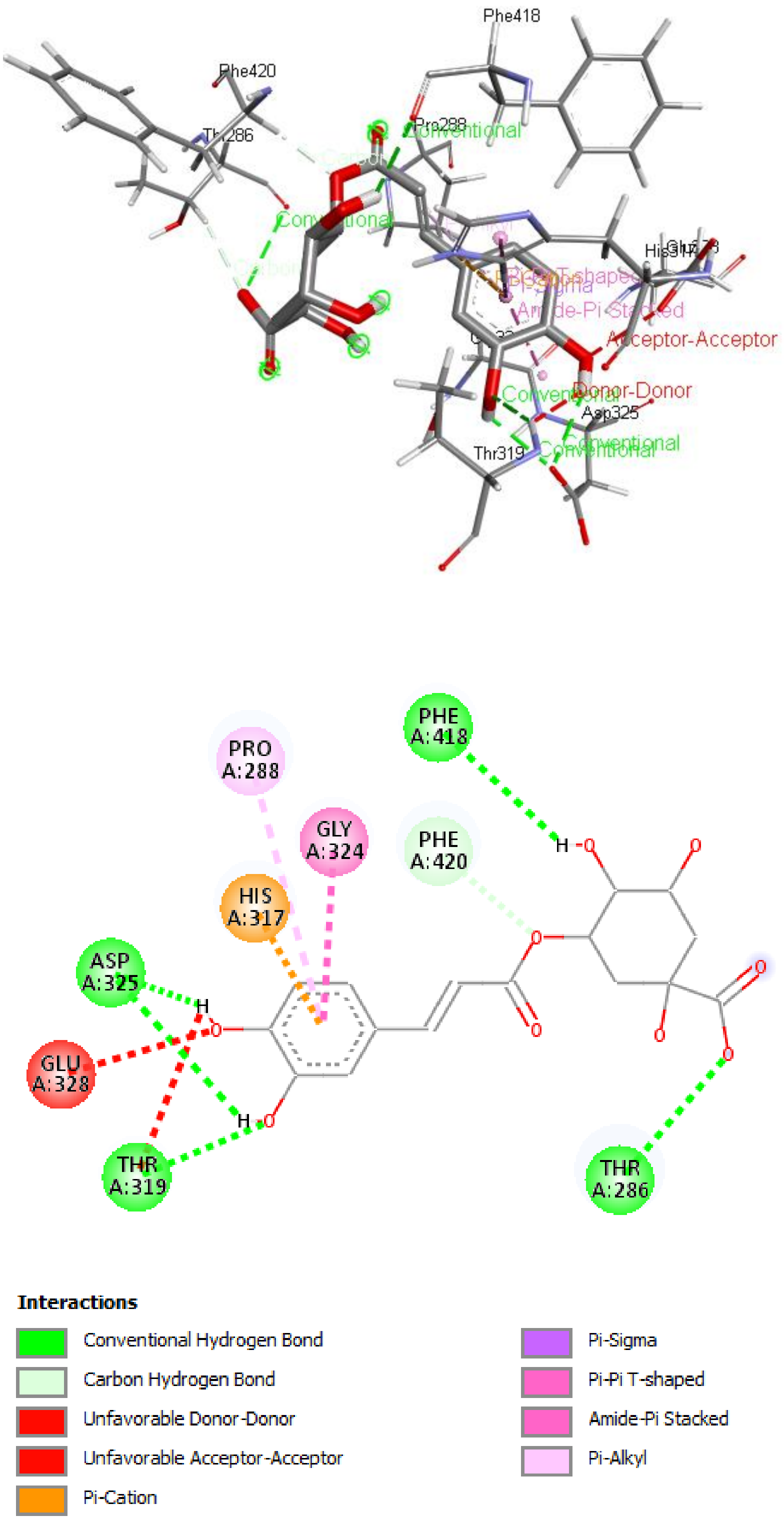

Figure S3. Interaction between Chlorogenic acid and Polyketide enzyme.

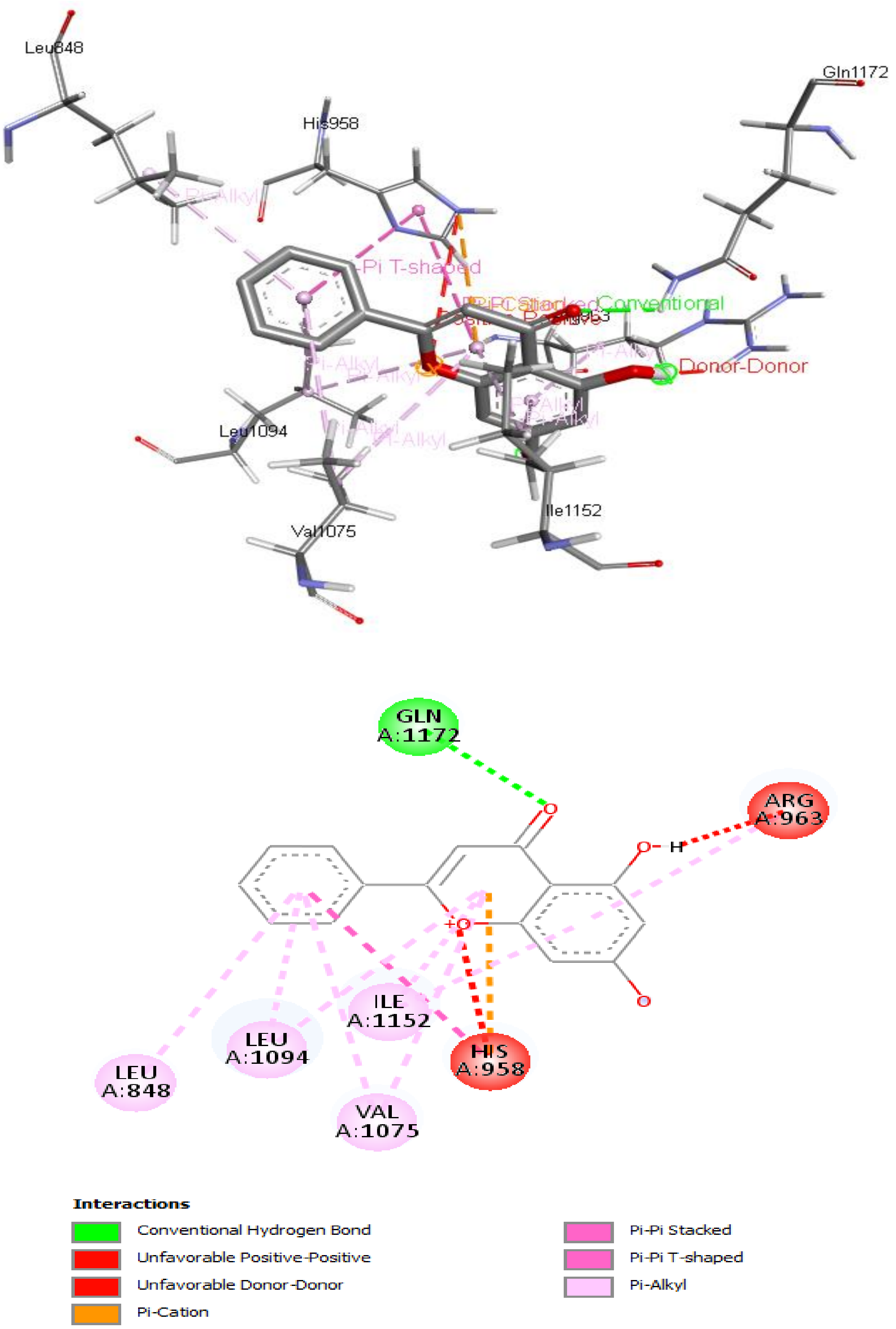

Figure S4. Interaction between Chrysin phenolic compound and non-ribosomal enzyme.

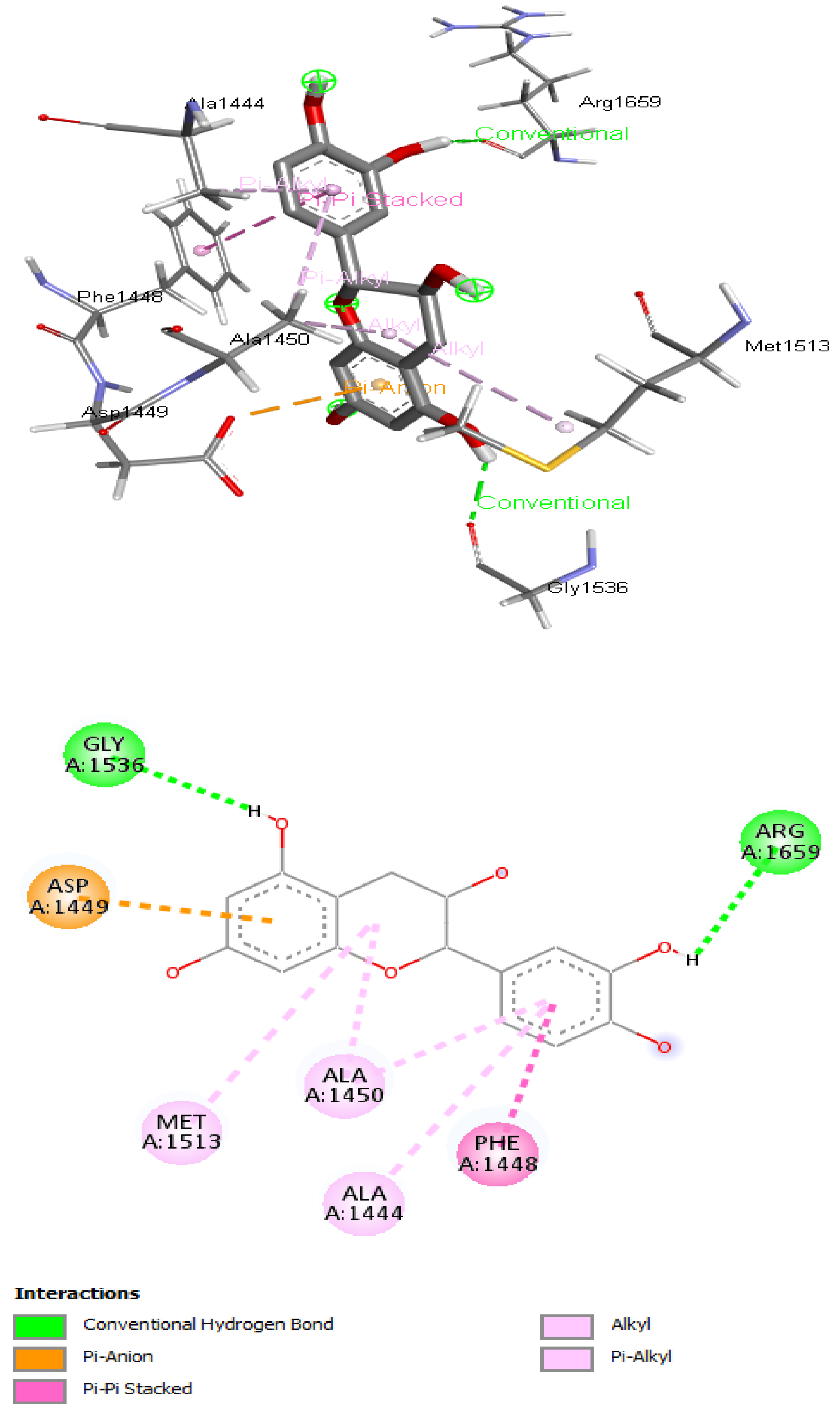

Figure S5. Interaction between catechin phenolic compound and non-ribosomal enzyme.
